# Postural control among elderly women with and without osteoporosis: is there a difference?

**DOI:** 10.1590/S1516-31802010000400009

**Published:** 2010-07-01

**Authors:** Thomaz Nogueira Burke, Fabio Jorge Renovato França, Sarah Rúbia Ferreira de Meneses, Viviam Inhasz Cardoso, Rosa Maria Rodrigues Pereira, Camille Figueredo Danilevicius, Amélia Pasqual Marques

**Affiliations:** I MSc student. Department of Physical Therapy, Speech and Occupational Therapy, Universidade de São Paulo (USP), São Paulo, Brazil.; II Student, Department of Physical Therapy, Speech Therapy and Occupational Therapy, Universidade de São Paulo (USP), São Paulo, Brazil.; III MD, PhD. Associate professor, Division of Rheumatology, School of Medicine, Universidade de São Paulo (USP), São Paulo, Brazil.; IV MD. Rheumatologist, Division of Rheumatology, School of Medicine, Universidade de São Paulo (USP), São Paulo, Brazil.; V MD, PhD. Associate professor, Department of Physical Therapy, Speech Therapy and Occupational Therapy, Universidade de São Paulo (USP), São Paulo, Brazil.

**Keywords:** Aged, Osteoporosis, Postural balance, Posture, Kyphosis, Idoso, Osteoporose, Equilíbrio postural, Postura, Cifose

## Abstract

**CONTEXT AND OBJECTIVE:**

Little is known about postural control among elderly individuals with osteoporosis and its relationship with falls. It has been suggested that elderly women with kyphosis and osteoporosis are at greater risk of falling. The aim of this study was to evaluate posture and postural control among elderly women with and without osteoporosis.

**DESIGN AND SETTING:**

Cross-sectional study conducted at the Physical Therapy and Electromyography Laboratory, School of Medicine, Universidade de São Paulo (USP).

**METHODS:**

Sixty-six elderly women were selected from the bone metabolism disorders clinic, Division of Rheumatology, USP, and were divided into two groups: osteoporosis and controls, according to their bone mineral density (BMD). Postural control was assessed using the Limits of Stability (LOS) test and the Modified Clinical Test of Sensory Interaction and Balance (CTSIBm) and posture, using photometry.

**RESULTS:**

The elderly women with osteoporosis swayed at higher velocity on a stable surface with opened eyes (0.30 versus 0.20 degrees/second; P = 0.038). In both groups, the center of pressure (COP) was at 30% in the LOS, but with different placements: 156° in the osteoporosis group and 178° in the controls (P = 0.045). Osteoporosis patients fell more than controls did (1.0 versus 0.0; P = 0.036).

**CONCLUSIONS:**

The postural control in elderly women with osteoporosis differed from that of the controls, with higher sway velocity and maximum displacement of COP. Despite postural abnormalities such as hyperkyphosis and forward head, the COP position was posteriorized.

## RESUMO

**CONTEXTO E OBJETIVO:**

Pouco se sabe sobre o controle postural de idosos com osteoporose e sua relação com as quedas. Foi sugerido que idosas cifóticas com osteoporose têm maior risco de quedas. Esta pesquisa teve como objetivo avaliar o controle postural e a postura em idosas com e sem osteoporose.

**TIPO DE ESTUDO E LOCAL:**

Estudo transversal realizado no Laboratório de Avaliação Fisioterapêutica e Eletromiografia da Faculdade de Medicina da Universidade de São Paulo (USP).

**MÉTODOS:**

Sessenta e seis mulheres idosas foram selecionadas da Clínica de Doenças Osteometabólicas da Divisão de Reumatologia da Universidade de São Paulo e divididas em dois grupos: osteoporose e controle, de acordo com a densidade mineral óssea (DMO). Foi avaliado o controle postural pelos testes Limite de Estabilidade (LOS) e Modified Clinical Test of Sensory Interaction and Balance (CTSIBm) e a postura pela fotometria.

**RESULTADOS:**

As idosas com osteoporose oscilaram com maior velocidade em superfície firme com olhos abertos (0,30 × 0,20 graus/segundo, P = 0,038). O COP (centro de pressão) de ambos os grupos encontrava-se a 30% do LOS, porém com posicionamentos distintos: 156° no grupo osteoporose e 178° no grupo controle (P = 0,045). As osteoporóticas caíram com maior frequência em comparação aos controles (1,0 × 0,0, P = 0,036).

**CONCLUSÃO:**

O controle postural de idosas com osteoporose diferiu dos controles, com maior velocidade de oscilação e máximo deslocamento do COP, e que apesar da presença de alterações posturais como hipercifose e anteriorização de cabeça, o COP se encontrou posteriorizado.

## INTRODUCTION

Osteoporosis is a common disorder characterized by reduced bone mass and by deterioration of the microarchitecture of the bone tissues, thereby leading to increased bone fragility.^1^ It affects around 55% of the population over the age of 50 years in the United States.^2^

Postural control is the inherent ability to maintain the center of mass on a supporting base, between stability limits. These limits are the operational areas up to which the center of mass can be displaced without the need to change the supporting base.^3^ Thus, balance depends on the individual’s ability to maintain postural control under a great variety of conditions, as well as the ability to perceive the stability limits.^4^ In order to avoid falling, the center of body mass must be kept within the supporting base or, even better, within the stability limits.

It has been suggested that elderly people present reduced ability to control their posture, which may predispose them to increased risk of falling.^5^ According to Jonson,^6^ age-related deterioration of balance or postural control has a negative impact on the ability to safely carry out day-to-day activities.

Among the likely causes of postural instability among the elderly, changes in the relationship between sensory information and motor action are of importance. The elderly have greater difficulty in interpreting sensory information and prioritizing it according to its relevance, and in selecting the proper response in order to maintain their balance in specific positions.^7^

Little is known about postural control among elderly individuals with osteoporosis and its relationship with falls. Lynn^8^ suggested that elderly women with kyphosis and osteoporosis were at greater risk of falling. This author suggested that the changes to the body caused by osteoporosis would displace the center of pressure (COP) closer to the limit of stability, thereby making it easier to lose balance, with consequent falls. However, Lynn’s study only assessed six women with osteoporosis and five controls, and the participants differed in age substantially (ranging from 52 to 85 years). Furthermore, although that study suggested that thoracic hyperkyphosis and forward head position gave rise to falls, these parameters were not objectively and quantitatively assessed.

Studying osteoporosis among the elderly is particularly important, since this group is at greater risk of developing fractures and comorbidities associated with falls. It has been estimated that for every decrease of one standard deviation of bone mineral density (BMD) in the head of the femur, there is a proportional 2.6-fold increase in the risk of fractures in the hip.^9^

## OBJECTIVE

Since few studies have measured postural control among elderly individuals with osteoporosis,^8,10^ and these studies did not measure the COP quantitatively, our aim here was to conduct a study to investigate postural control and posture among elderly women with and without osteoporosis. We hypothesized that women with osteoporosis would present diminished postural control and an anteriorly shifted COP, caused by postural abnormalities such as hyperkyphosis and forward head, in relation to elderly individuals without osteoporosis.

## METHODS

### Sample characteristics

Our sample consisted of 66 women with ages ranging from 66 to 81 years. They were recruited from the bone metabolism diseases outpatient clinic of the Division of Rheumatology, Universidade de São Paulo (USP). The participants were divided into two groups, according to their BMD: the osteoporosis group (n = 46) presented BMD that was at least 2.5 standard deviations (SD) lower than the standard values for young adults, in relation to the lumbar spine, femoral neck and total femur regions.^11^ The control group (n = 20) presented BMD that was above -2.5 SD in relation to the same areas.

**Figure 1.**
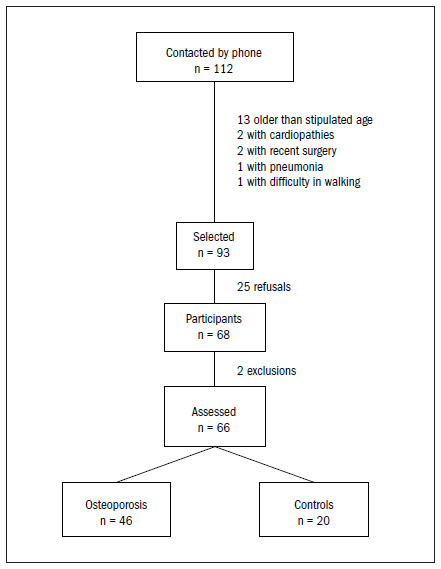
Flow of the study.

Patients were excluded if they presented significant visual impairment; inability to walk more than 10 meters without assistance; neurological or musculoskeletal diseases (e.g. Parkinson’s disease, stroke or neurodegenerative disorders); or amputations and prostheses for the arms or legs. Figure 1 displays the flow of our study. The participants signed an informed consent form. The study and consent forms were approved by the Ethics Committee of Hospital das Clínicas (HC) of the USP School of Medicine.

### Measurements

#### History of falls

A blinded investigator (i.e. blinded to group status) applied a questionnaire in order to obtain information on age, weight, height and history of falls over the past year. Falls were defined as non-intentional contact of hands, arms, chest or hips with the floor, after losing balance.

#### Postural control

Postural control was assessed using the Modified Clinical Test of Sensory Interaction and Balance (CTSIBm) and the 100% Limits of Stability (LOS) test. The CTSIBm measures static equilibrium under four sensory conditions: stable surface and opened eyes (SS-OE); stable surface and closed eyes (SS-CE); unstable surface and opened eyes (US-OE); and unstable surface and closed eyes (US-CE).

In order to pick up the COP sway velocity (degrees per second), we used a force platform (model: NeuroCom Balance Master). The COP indicated the location of the vector resulting from the reaction force applied on the ground, as measured by the force platform. This vector is the same as (but opposite in direction to) the weighted mean of all the forces acting on the force platform, such as weight and the internal forces (from the muscles and joints) that are transmitted to the ground.^12^ Each experiment was repeated three times, for 10 seconds; we have presented the means from the experiments. While performing the CTSIBm, we also measured the position of the COP in relation to the center of the ellipse in the LOS test. The results have been presented as percentages of LOS and in degrees.

**Figure 2.**
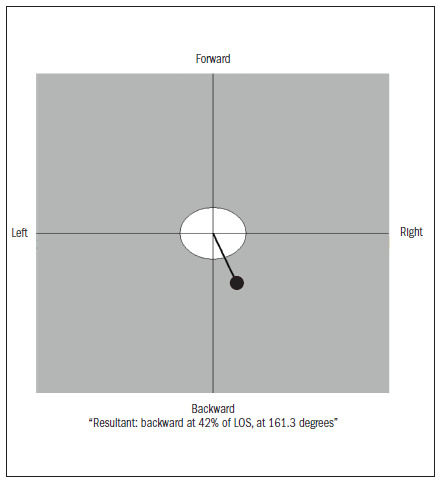
Example of positioning the center of pressure (COP) in relation to the center of the ellipse of the limits of stability.

**Figure 3.**
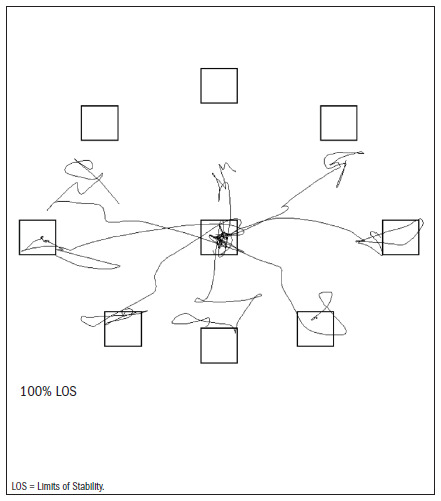
Example of the 100% limits of stability test.

During the 100% LOS test, the participants had to reach out to touch eight different targets that were distributed symmetrically around a central point that represented the maximum distance (theoretical LOS) through which the subjects would be able to shift their COP without losing balance (Figure 2), and without moving their feet (Figure 3). We defined the variable of maximum excursion as the greatest distance reached out towards the targets by the center of gravity at any point during the attempts. This was expressed as a percentage of the theoretical LOS.^13^ We decided to merge all shifts in the anterior-posterior and side-to-side directions.

For the CTSIBm and 100% LOS tests, the participants remained in the orthostatic position, with arms extended along the sides of the body, without wearing shoes. They were placed in one of the three standard positions recommended by the manufacturer.

#### Posture

Posture was assessed by means of photometry, which consisted of capturing images using a digital camera for subsequent analysis of anatomical points that had previously been marked out. We used a specifically designed postural assessment software: *Software de Avaliação Postural* (SAPO), available at www.sapo.incubadora.fapesp.br.^14^

Anatomical points were marked on the skin using markers of 15 mm in diameter. Photographs were taken with the individuals minimally dressed, such that it was possible to view the following anatomical points: tragus of the ear, 7^th^ cervical vertebra, 12^th^ thoracic vertebra and midpoint of the acromion. From analyzing the points and their relationships, we made measurements relating to the conditions of forward head and thoracic kyphosis.

The head position was determined in terms of the angle between the vertical line passing through the midpoint of the acromion and the line drawn between the midpoint of the acromion and the tragus.^15^ Positive values signified that the tragus was advanced in relation to the acromion and, therefore, a forward head position; negative values indicated retraction of the head.

The measurements of thoracic kyphosis were based on the kyphosis index, as assessed using the flexicurve method proposed by Takahashi and Atsumi,^16^ with the modifications suggested by Teixeira and Carvalho.^17^ The results were presented in degrees. This method presents high intraclass correlation (ICC = 0.906), in comparison with kyphosis measurements using Cobb’s angle.^17^ Thoracic hyperkyphosis was diagnosed when the angles were greater than 50º, as described by Wilner.^18^

In order to be photographed, the participants remained in the orthostatic position, with their feet in the position recommended by the manufacturer of the equipment. To facilitate this procedure, we manufactured a rug with the same dimensions and marks as on the force platform. The camera was positioned at a distance of two meters from the participants, and at an elevation of one meter above ground level, in the horizontal plane, i.e. parallel to the plane that was to be observed.^19^ A ribbon guide was placed on the individual, in the same plane. All the images were taken with the camera, tripod, ribbon guide and patient in the same positions. We used a Sony Cybershot P-92 digital camera, and images were transferred to the postural analysis software (SAPO) to make the assessments of interest.

### Statistical analyses

The data were compared using the SigmaStata 3.5 software. The sample size was determined by taking the power to be 80% and the standard deviation and expected difference in means to be 20%, with α = 0.05. Thus, a minimum number of 17 subjects per group was determined. We used the t test for parametric variables such as age, weight, body mass index (BMI), BMD (total femur), COP position (%LOS) and maximum COP excursion COP. The results were presented as means and standard deviations. For non-parametric variables, such as BMD (lumbar spine), COP location (degrees), falls and COP velocity, we used the Mann-Whitney test, and medians were presented. The significance level was established as 5% (α = 0.05), with 95% confidence intervals.

## RESULTS

The anthropometric and demographic characteristics of the 66 individuals are described in Table 1. The participants were non-institutionalized elderly women (age range: 66-80 years), who were able to walk independently. The two groups were similar in terms of age. The individuals with osteoporosis presented significantly lower weight, and height, in comparison with the controls. The individuals in the osteoporosis group reported significantly more falls than did those in the control group (P = 0.036).

**Table 1.** Anthropometric and demographic characteristics of individuals with osteoporosis and controls

Variable	Osteoporosis (n = 46)	Control (n = 20)	P-value
Age, *years*	73.0 (4.2)	71.9 (3.2)	0.262
Weight, *kg*	57.7 (8.6)	68.4 (7.3)	0.000
Height, *m*	1.50 (0.06)	1.55 (0.06)	0.003
BMI, kg/*m*^2^	25.6 (3.4)	28.3 (2.4)	0.002
BMD, T-score*			
Lumbar spine	-3.5	-1.0	0.000
Total femur	-2.1 (0.6)	-0.4 (0.8)	0.000
Falls/patient†, n	1.0	0.0	0.036

* BMD = bone mineral density, as compared with individuals with maximum bone density;

† preceding year;. Values lower than -2.5 standard deviations suggest osteoporosis.

In the osteoporosis group, 70% of the individuals had thoracic hyperkyphosis, versus 40% among the controls. Table 2 displays the values for postural control among the individuals with and without osteoporosis. The individuals with osteoporosis presented higher sway velocities under all four conditions tested by the CTSIBm. However, these differences only reached significance in the tests on a stable surface with opened eyes (P = 0.038). In the LOS test, the individuals with osteoporosis presented significantly higher amplitudes of displacement than observed among the controls, when asked to reach their LOS. The difference was significant when the displacement was in the anterior-posterior direction, but not in the side-to-side direction. The COP was placed at the same percentage as the LOS, but with different angulation (P = 0.045).

**Table 2.** Postural control among individuals with osteoporosis and in controls

Variable	Osteoporosis (n = 46)	Control (n = 20)	P-value
CTSIBm, COP velocity (°/s)*			
SS-OE	0.30	0.20	0.03^†^
SS-CE	0.30	0.30	0.32
US-OE	1.05	1.00	0.12
US-CE	3.30	3.10	0.34
COP position			
Degrees*	156.5	178.3	0.04^†^
% LOS	28.0 (15.9)	32.9 (12.8)	0.22
LOS Maximum excursion, *% LOS*			
Anterior-posterior	69.9 (14.0)	57.2 (17.4)	0.003†
Side-to-side	101.8 (14.8)	94.6 (18.4)	0.09
Posture			
Forward head,*degrees*	19.0 (13.1)	14.4 (8.6)	0.16
Kyphosis, *degrees*	53.1 (13.8)	45.9 (10.1)	0.05†
Hyperkyphosis, *n* (%)	70%	40%	

* Median values; SS-OE = stable surface and opened eyes;

† Statistically significant difference between the groups (P < 0.05); SS-CE = stable surface and closed eyes; US-OE = unstable surface and opened eyes; US-CE = unstable surface and closed eyes.

## DISCUSSION

The aim of this study was to assess posture and postural control among women with and without osteoporosis. The elderly women with osteoporosis presented higher sway velocity and higher numbers of falls, in comparison with the controls. When asked to shift their COP closer to their stability limits, the women with osteoporosis had higher sway amplitudes in the anterior-posterior direction, in comparison with the controls. Furthermore, among the women with osteoporosis, the COP was displaced posteriorly and in the right lateral direction; among the controls, the COP was displaced posteriorly. Both groups showed forward head and thoracic hyperkyphosis: the proportions were different (osteoporosis group = 70%; controls = 40%), but this difference was not significant.

According to Hageman et al.,^20^ postural sway in the vertical position seems to increase with age, and this is prompted when the support base is modified (through decreasing its size or changing its surface to a foam surface), the body configuration is changed (standing on one foot) or the visual input is changed. This happens because postural control depends on harmony between the visual, vestibular, proprioceptive and musculoskeletal systems.^21^

We found that the sway velocity among the women with osteoporosis was 16.7% higher than among the controls. Our findings are in agreement with those of Liu-Ambrose et al.^22^, who found balance scores that were 11% lower among women with osteoporosis. Considering the four different conditions of our test, the greatest difference was seen in relation to the stable surface and opened eyes, in which the women with osteoporosis swayed at a velocity that was 50% greater than shown by the controls. Under the remaining conditions (stable surface with closed eyes and unstable surface with opened or closed eyes), the women with osteoporosis also swayed at higher velocity, but the differences were not significant.

It is important to emphasize that greater differences between the groups were seen when the eyes were opened. This may reflect a "ceiling effect", caused by the additional difficulties when performing the tasks with closed eyes (reduced discriminatory properties of the test). In situations of increased difficulty (e.g. eyes closed or unstable surfaces), the differences decreased and both groups swayed at a higher velocity.

Our data suggest that the individuals with osteoporosis displaced the COP with a higher amplitude in the anterior-posterior direction, in comparison with the controls. This finding was unexpected, since voluntarily shifting the COP towards the stability limit depends on lower-limb muscle strength, along with the trust that individuals have in their own ability to move. Studies have suggested that individuals with osteoporosis present reduced strength and greater fear of falling.^3,23,24^

Specifically focusing on our findings, it may be that the lower weight (8.1 kg lower) and BMI (3.3 kg/m^2^ lower) relative to the controls, which is characteristic of individuals with osteoporosis,^25,26^ may have had an influence. Weight may have negatively influenced the ability to displace the COP. This behavior was less evident in the side-to-side direction. Era et al.^27^ described an association between low BMI and deficient balance among elderly individuals, thus supporting our findings.

Melzer et al.^5^ suggested that decreased postural control may be a risk factor for falls among the elderly, and that the impairment is probably caused by conflicting sensory-motor inputs. Through such conflicts, elderly people would have greater difficulty in identifying the most relevant sensory information, and in developing the proper postural reaction to maintain their balance in the desired position.^7^

Both groups presented forward head posture and increased thoracic kyphosis, which are typical of the elderly. According to Lynn et al.,^8^ a kyphotic posture displaces the center of gravity towards the anterior stability limit, thereby requiring increased effort in order to maintain balance, even after minor changes. In this regard, our study yielded contradictory findings, since both groups presented the COP displaced posteriorly and to the right, at 30% of the LOS. Among the individuals with osteoporosis, it was displaced to the right (156º), while among the controls, it was displaced posteriorly (178º). It may be that displacement of body structures caused simply by a kyphotic posture and by a forward head posture are insufficient to disrupt balance, and to shift the COP towards the stability limit.

The only two postural parameters that we assessed were forward head and thoracic kyphosis. However, compensatory displacements of other structures, such as hip antepulsion and trunk extension, may have influenced the final position of the COP, since it is determined not only by the sum of the segmental weights but also by their spatial positions.^10^ Our findings support the concept that posture should not be analyzed segmentally, but in an overall manner. It is important to observe the relationships between all segments in the body, since compensatory displacements influence the COP and, therefore, body equilibrium. Accordingly, future larger studies with overall assessment of posture are need in order to confirm or refute our findings.

## CONCLUSIONS

Our study suggests that postural control among individuals with osteoporosis is different from postural control among the general elderly population. Individuals with osteoporosis are more likely to present higher sway velocities and greater maximum shift of the COP. Despite postural abnormalities such as forward head and kyphosis, the COP is located posteriorly. Better understanding of the determinants of the COP among elderly individuals with osteoporosis, as well as of its relevance in relation to causing falls, is of importance in order to develop preventive strategies with the aims of improving quality of life and reducing comorbidities among the elderly.
